# On the Therapeutical Properties of Iodine

**Published:** 1846-08

**Authors:** J. K. Mitchell

**Affiliations:** Professor of the Practice of Medicine in Jefferson Medical College, etc.


### MEDICAL EXAMINER,

AND

### RECORD OF MEDICAL SCIENCE.

NEW SERIES.—No. XX.—AUGUST, 1846.

### ORIGINAL COMMUNICATIONS.

*On the Therapeutical Properties of Iodine.* By J. K. Mitchell, M. D., Professor of the Practice of Medicine in Jefferson Medical College, etc.

Perhaps no medicine has been so much praised and condemned as iodine and its preparations. While some practitioners depend almost indiscriminately on its remedial efficacy, others, of not less reputation and experience, declare that they have yet to see one case in which it has unequivocally manifested any useful property. Some, indeed, have asserted that iodine in any form is only hurtful, having power only for evil. Destroyed mucous tissues, obstructed glands, incurable chronic gastritis, and intense and unmanageable cephalalgia are a few of the mischievous effects alleged to belong to the use of iodine. Its advocates, on the other hand, give apparently enormous doses without any apprehension of injurious consequences, and boldly declare that nothing but good has resulted from their administration.

My own observation and frequent conferences with many judicious physicians, induce me to place myself in an intermediate position, and to assume the ground that iodine is not only a useful article of the materia medica, but a very great addition to our means of cure. I am, however, far from believing that very large doses are either necessary or hurtless, as I have not unfrequently witnessed very distressing effects not only from an extravagant quantity, but from the too prolonged use of even moderate portions of it.

That free iodine is an active poison few will deny; but there are many who seem to think iodide of potassium perfectly innoxious, and therefore they apply it not only in any dose, but to almost every malady. Though not justified by uniform success or unqualified safety, these large doses are given so often and so long, as to show that while there is hazard and injury, the danger has been greatly overrated.

The first writer of much repute on iodine was Lugol, who thought that it seldom acted advantageously unless given in minute doses and for a very prolonged period of time. He also thought that only free iodine was of much advantage to the patient, the other preparations only proving useful by such decompositions as extricated it. More recent observations show that iodine acts sometimes with surprising celerity and to the production of astonishing effects, and under circumstances which do not sustain Lugol's theory of its action.

My present purpose is to recite some such cases, not doubting that most of my professional brethren have seen similar ones, because their frequency in my own sphere of observation, and cases already published by others, warrant the supposition.

Mr. M—— N——, S. Fifth street, Philadelphia, was seen by me for the first time on Monday evening in June, 1845. I found him confined to bed by a very painful affection of the head and neck—with occasional and pungent pain of the arms and legs. The superficial arteries of the temples were turgid—the expression anxious and sad—the skin puffy and shining. For several weeks he had not slept more than half an hour at any one time, and seldom more than one hour in the day. For upwards of six months he had not been able to attend to business, and for nearly five years his life had been embittered by neuropathic maladies. He had tried the usual and most of the unusual methods of alleviating pain, and had gone the round of the several empirical systems which promise relief to the sufferer. Despite them all his pains grew worse, and, as he said, nothing gave even temporary alleviation. Even the extraction of almost all his teeth had failed to afford him a respite from pain.

On enquiry I discovered that he had not used any preparation of iodine, although he had encountered arsenic, mercury and most of the narcotics. I therefore ordered him a solution of iodide of potassium, so that he might take seven grains three times a day. Business took me to New York. On my return after eight days, I was agreeably surprised to learn that on the second night the patient had slept well for two or three hours, still better on the third, and on the fourth day perceived an improvement in his appetite and an increase in his locomotive and intellectual faculties. On the seventh day he was able to visit some of his friends. On this day he was weighed, and perceived that he had lost within two years nearly 75 lbs. From this time he ceased to feel any pain, and gradually recovered his health, strength, appetite and weight. Within six weeks every vestige of his malady and its results were obliterated.

Mr. S——, a respectable merchant, residing in Vine street above Tenth, consulted me in the winter of 1842-3—about the first of January. He had been afflicted with erratic pains for nearly five years, and became at last so sore as to be unable to walk with comfort. He had not been out of his house for three months, and had lost his power of sleeping, as well as of digesting correctly his food. Flatulent dyspepsia disturbed his slumbers, and his arms, bent and painful, would not permit him "to spread the butter on a slice of bread."

I left with him the following prescription:

℞. Iod. potass. ℥ss.

Iodin. grs. x.

Aquæ Oss. M.

Sig. To take a teaspoonful in cold water three times a day.

I believed, and so told him, that he would be relieved within a month, and would probably be able to visit me then at my office.

On the twelfth day after my first visit, Mr. S. came into my office, erect and active, entirely free from pain, without flatulency, stiffness or any other apparent ailment. He was well; and has so continued up to the present time. He perceived a sensible diminution of pain and rigidity in less than forty-eight hours after the administration of the remedy.

Mr. A——, a very respectable cabinet maker, presented an enormous tumefaction, not only of the thyroid gland, but of the lymphatic glands of the neck—so that as his head was somewhat conical near the vertex, and his neck extended nearly to the breadth of his shoulders, he represented a rude model of a sugar loaf, the base resting on the shoulders. A skilful surgeon pronounced the case hopeless, beyond the reach of the knife, and for which he had no other remedy. He, however, at the earnest entreaty of the patient, put in requisition the usual depletion and exutories, with mercury and cicuta. The case grew worse—severe dyspnœa supervened, deglutition became difficult, and death seemed at hand. We proposed then to try iodine, a remedy of which at that time neither of us had much direct knowledge. Within a single day, the patient breathed better and became thoroughly convinced of approaching recovery; and, at the end of a week, experienced very little inconvenience from, though still deformed by his malady. In a few months the thyroid no longer hung over his sternum—not an abnormal gland could be felt in his neck—and he recovered both his health and personal appearance entirely.

The mastery over pain exercised under certain conditions by this singular remedy, was curiously manifested in the case of Mr. McC., a reputable farmer in this vicinity, who consulted Dr. Mütter and myself for a tumour of large size and schirrous character, seated in the neck and protruding into the *isthmus faucium.* Although the tumour impeded deglutition and respiration, and much disfigured the patient, he seemed to suffer chiefly from a prolonged ceaseless pain of an aching and depressing character. Not hoping for more than a temporary benefit, we endeavoured by diet, opiates and local depletion, to lessen the size of the tumour and the sufferings of the patient—but in vain. Iodide of potassium in eight grain doses was then ordered. After the second dose the pain ceased for some days, although the tumour continued to enlarge. The pain returned at irregular periods, but was always, as speedily as at first, relieved by iod. of pot., and the patient died of dyspnœa, perfectly conscious to the last, *and free from pain.*

My case-book contains several similar examples, but as they do not offer any peculiarities, it is scarcely necessary to recite them.

In my next communication I will endeavour to show some of the evils which result from the indiscriminate use and excessive administration of iodine. Before closing this hastily written paper, I may remark that tumours yield usually most readily to the direct or endermic application of iodine as a tincture or unguent, and that bronchocele is peculiarly under its influence when thus applied.

